# Impact of random safety analyses on structure, process and outcome indicators: multicentre study

**DOI:** 10.1186/s13613-017-0245-x

**Published:** 2017-02-28

**Authors:** María Bodí, Iban Oliva, Maria Cruz Martín, Maria Carmen Gilavert, Carlos Muñoz, Montserrat Olona, Gonzalo Sirgo

**Affiliations:** 1 Intensive Care Unit, Hospital Universitario Joan XXIII, Tarragona, Spain; 2 Instituto de Investigación Sanitaria Pere Virgili, Rovira i Virgili University, Tarragona, Spain; 3 Centro de Investigación Biomédica en Red de Enfermedades Respiratorias (CIBERES), Instituto de Salud Carlos III, Madrid, Spain; 4 Intensive Care Unit, Hospital Universitario de Torrejón, Torrejón de Ardoz, Madrid, Spain; 5 Department of Preventive Medicine, Hospital Universitario Joan XXIII, Tarragona, Spain

**Keywords:** Safety, Intensive care unit, Critical patients, Real-time safety audits, Quality indicators

## Abstract

**Background:**

To assess the impact of a real-time random safety tool on structure, process and outcome indicators.

**Methods:**

Prospective study conducted over a period of 12 months in two adult patient intensive care units. Safety rounds were conducted three days a week ascertaining 37 safety measures (grouped into 10 blocks). In each round, 50% of the patients and 50% of the measures were randomized. The impact of this safety tool was analysed on indicators of structure (safety culture, healthcare protocols), process (improvement proportion related to tool application, IPR) and outcome (mortality, average stay, rate of catheter-related bacteraemias and rate of ventilator-associated pneumonia, VAP).

**Results:**

A total of 1214 patient-days were analysed. Structure indicators: the use of the safety tool was associated with an increase in the safety climate and the creation/modification of healthcare protocols (sedation/analgesia and weaning). Process indicators: Twelve of the 37 measures had an IPR > 10%; six showed a progressive decrease in the IPR over the study period. Nursing workloads and patient severity on the day of analysis were independently associated with a higher IPR in half of the blocks of variables. Outcome indicators: A significant decrease in the rate of VAP was observed.

**Conclusions:**

The real-time random safety tool improved the care process and adherence to clinical practice guidelines and was associated with an improvement in structure, process and outcome indicators.

**Electronic supplementary material:**

The online version of this article (doi:10.1186/s13613-017-0245-x) contains supplementary material, which is available to authorized users.

## Background

The application of evidence-based medicine is of major concern in intensive care medicine today [1]. Errors in health care may occur due to an unintended act or by omission. Those resulting from the former are more visible and therefore more easily detectable. Errors of omission are more insidious and more difficult to identify and include the failure to ensure that patients receive recommended medical care as supported by high-quality clinical research evidence [2], which occurs paradoxically in more severe patients [3]. For example, the lack of adherence to clinical practice guidelines may be due to the lack of knowledge about them and the presence of barriers that prevent their use such as a lack of time, a lack of resources, organizational aspects or even resistance to changing work habits.

To analyse and prevent patient safety-related incidents, reactive or proactive tools are used. These are complementary to each other. Checklists have been proposed as a simple and useful proactive method to prevent errors of commission and omission in critically ill patients [4, 5]. The complex reality in which they need to be implemented requires an approach that includes more than eliminating barriers and supporting facilitating factors. Implementation leaders must facilitate team learning to foster the mutual understanding of perspectives and motivations and the realignment of routines [6].

Among the various proactive methods, random safety audits [7] facilitate the interaction between the responsible team and the professional who verifies the safety measures, and have the potential to reduce future errors through the identification of system failures that contribute to gaps in quality and safety. This tool promotes the changes in accordance with the application of scientific evidence, feedback with the team, and providing and strengthening knowledge [8]. Weiss et al. [9] showed that checklists of safety measures guided by an observer (prompter) decreased mortality and average length of stay in an intensive care unit (ICU) compared to those carried out through self-verification.

Our group has developed and validated a tool—the real-time random safety audits (in Spanish: Análisis Aleatorios de Seguridad en Tiempo Real, AASTRE)—and found it to be effective in detecting and remedying errors of omission in real time, thereby improving adherence to guidelines [10] and proving to be most useful in situations of high care load and in more severe patients [11].

Thus, this multicentre study aims to investigate the usefulness of the AASTRE by measuring their effect on structure, process and outcome indicators.

## Methods

### Study design and participating centres

This is a prospective study involving two university hospitals over a 1-year period (January–December 2013). Table 1 shows the characteristics of the two centres and the most relevant initiatives implemented in terms of patient safety.

**Table 1:** Characteristics of the centres and safety-related initiatives

	Hospital 1	Hospital 2
No. of hospital beds	250	350
Teaching hospital		
Undergraduate	Yes	Yes
Resident physician	No	Yes
No. of ICU beds		
Total at the centre	16	30
ICU participating in study	16	14
Computerized ICU	Yes	Yes
Active protocol for:		
Sedation and analgesia	No	Yes
Weaning	No	Yes
Enteral nutrition	Yes	Yes
Monitoring and MV alarms	No	No
Register of AE	No	No
Voluntary reporting of AE	Yes	Yes
ENVIN-ICU participation (BZ, NZ, RZ)	Yes	Yes
Other checklist systems (Not AASTRE)	Prevention of VAP, CRB	Prevention of VAP, CRB, intrahospital transfer
Patient types	Medical	Medical, surgical, trauma
	Surgical	
	Trauma	
	Coronary	

*No.* absolute number, *ICU* intensive care unit, *MV* mechanical ventilation, *AE* adverse events, *ENVIN* (National Nosocomial Infections Surveillance Study), *BZ* Bacteremia Zero Spanish Project, *NZ* Pneumonia Zero Spanish Project, *RZ* Resistance Zero Spanish Project, *VAP* ventilator-associated pneumonia, *CRB* catheter-related bloodstream infection

### Methodology for the implementation of the AASTRE

#### Design and description of the checklist

The checklist, as previously validated [10], consists of 37 safety measures grouped into ten blocks of different areas of care: mechanical ventilation, haemodynamics, renal function and continuous renal replacement techniques (CRRT), sedation and analgesia, treatment (two blocks), nutrition, techniques and tests, nursing care and structure. AASTRE are standardly performed three days per week (including weeks with weekday holidays and holiday periods), with 50% of the safety measures and 50% of the ICU patients randomized on each day of analysis. Each safety measure has a specific definition, assessment criteria and a specific methodology for verification. All patients admitted to the ICU are eligible for AASTRE to be performed on them. However, for each selected patient, only those measures for which they meet the assessment criteria will be evaluated [11].

#### Role and training of prompters

The safety audits are always carried out immediately after the ICU daily clinical round and require the participation of a prompter and the healthcare professionals directly responsible for patient care (senior attending physician, residents and nurses). The prompter is one of the two senior attending physicians of each ICU (not directly caring for the patient) who has received the education and training required by the study and who is responsible for verifying and/or promoting the safety measures. At all centres, training sessions were held on the theoretical aspects and the methodology used in the AASTRE. In addition, all prompters were trained online in the goals of the study and in the use of the tool. Moreover, practical training was also required, carrying out at least three safety rounds prior to the start of the study.

#### Safety audits

Many of the measures included in the checklist are routinely carried out by healthcare professionals during the ICU daily clinical round. The purpose of the safety audits is to verify that they have indeed been carried out. If this were not the case (error of omission), the prompter reminds the healthcare professionals that they should be carried out. In this framework, the possible responses during the audits are: (1) “Yes”—when the measure analysed had been taken/performed on the ICU daily round; (2) “Yes, after AASTRE”—when the safety audit was used to detect an error of omission that has been corrected; (3) “No”—when the measure analysed could not be changed despite the audit; (4) “Not applicable”—when the patient did not meet the assessment criteria. The checklist and the responses of the evaluations are entered into a web platform (http://www.aastre.es). Safety audits were performed with a tablet at the bedside to facilitate implementation.

### Definition of variables and indicators

1. *Number of patient*-*days* was the number of patients assessed in the total number of days on which safety audits were carried out in the two hospitals.
2. *Structure indicators*
  Perception of safety culture (in Hospital 2): We used a previously validated questionnaire [12] based on the Safety Climate Survey (SCS) and the Safety Attitude Questionnaire-ICU model (SAQ-ICU). It analysed six dimensions: teamwork climate, safety climate, perceptions of management, job satisfaction, working conditions, and stress recognition. The questionnaire on the perception of safety culture was administered to medical, nursing and ancillary staff. Three evaluation periods were considered: 1) initial period: the month prior to the start of the study; 2) intermediate period: month 6 of the study; 3) final period: the month after the end of the study.
  The execution or updating of protocols and/or procedures promoted by the AASTRE was investigated.
3. *Process indicators *
  The proportion of changes in the care process carried out as a result of verification was considered. IPR-AASTRE (improvement proportion related to the AASTRE) were calculated globally (IPR-AASTRE-G), for each safety measure (IPR-AASTRE), and for each block of variables (IPR-AASTRE-B), according to the following formulas:
    $$ {\text{IPR}} - {\text{AASTRE}} = \frac{{{\text{number of
occasions on which the AASTRE changed clinical practice }}\left(
{\text{{"yes}},     {\text{after the AASTRE"}}}
\right)}}{{{\text{number of occasions on which the measure was
selected }} - {\text{number of occasions on which the measure was
not applicable}}}} \times 100 $$
    $$ {\text{IPR}} - {\text{AASTRE}} - {\text{B}} = \frac{\text{sum of the number of occasions on which the AASTRE changed clinical practice in each block}}{{{\text{number of occasions on which the measure was selected in each block}} - {\text{number of occasions on which the measure was not applicable in each block}}}} \times 100 $$
    IPR-AASTRE-B helped simplify the assessment of the impact of other variables on utility. These variables are: type of patient (medical, surgical, neurocritical and trauma), staffing ratio [PNR; patient:nurse ratio (≤2:1 vs. >2:1) and PPR; patient:physician ratio (≤2:1, 2–3:1, >3:1)], the Sequential Organ Failure Assessment (SOFA) score and length of stay (length of stay at the time of safety audits (<7, 7–14, >14 days).

4. *Outcome indicators*
  The impact by the AASTRE on ICU mortality, average stay and rates of central venous catheter-related bacteraemia (CRB) and ventilator-associated pneumonia (VAP) using standardized definitions [13, 14] was investigated. The clinical definition of VAP requires patients to fulfil one radiographic, one systemic, and two pulmonary criteria. Radiographic criteria include new or progressive infiltrates, consolidation and cavitation. Systemic criteria include fever, abnormal white blood cell count and altered mental status. Pulmonary criteria include purulent sputum, new or worsening cough or dyspnoea or tachypnea, rales or bronchial breath sounds, and worsening gas exchange. CRB is defined in a patient with a central venous catheter with at least one positive blood culture (two blood culture if common skin contaminant organism) obtained from a peripheral vein, clinical manifestations of infections (i.e. fever, chills and/or hypotension), and no apparent source for the bloodstream infection except the catheter. One of the following should be present: a positive semi-quantitative (>15 CFU per catheter segment) or quantitative (>10^2^ CFU per catheter segment) catheter culture, whereby the same organism (species) is isolated from a catheter segment and a peripheral blood culture; simultaneous quantitative blood cultures with a ratio of >3:1 CFU/ml of blood (catheter vs. peripheral blood); differential time to positivity (growth in a culture of blood obtained through a catheter hub is detected by an automated blood culture system at least 2 h earlier than a culture of simultaneously drawn peripheral blood of equal volume). The information relative to VAP and CRB was collected prospectively at both centres participating in the study and the previous year, using identical diagnostic criteria.

### Statistical analysis

For descriptive analysis, we used absolute numbers (*N*) and relative frequency (percentage) for categorical variables; the mean and standard deviation for continuous variables. Chi-square tests and linear trend Chi-square tests were used for categorical variables and Student’s *t* test for continuous variables in univariate analysis. For multivariate analysis, multiple logistic regression, fixed model and likelihood ratio method analyses were performed to ascertain the impact of different variables on the IPR-AASTRE-B and with the aim of adjusting for possible confounding effects. The results were expressed as odds ratio and their 95% confidence interval (CI).

We used direct standardization by APACHE 2012 (<15, 15–25, >25) to evaluate mortality change and incidence density ratio (IDR) 2013 vs 2012 and CI to evaluate CRB and VAP incidence changes. The acceptable level of statistical significance was set at *p* ≤ 0.05. All data analyses were performed using the SPSS version 15 statistical package (SPSS Inc., Chicago, IL).

## Results

During the study period, AASTRE were carried out on 1214 patient-days. Table 2 shows the distribution of the type of patients evaluated globally, and in each hospital, the workloads (of nursing staff and physicians), the seriousness of the patients measured using the SOFA and patients’ length of stay on the day the safety rounds were conducted. Most patients were medical (47.0%), with a PNR ≤2:1 (57.0%), a PPR 2–3:1(62.1%), SOFA <4 (56.9%) and an average stay <7 days (42.5%). It should be noted that the distribution of the types of patients evaluated is different in the two hospitals of the study. In Hospital 1, there was a predominance of medical patients (57.6%), followed by surgical patients (32.7%). In Hospital 2, although the evaluation of medical patients predominated (41.3%), followed by surgical patients (31.6%), there was a significantly higher percentage of assessments of neurosurgical (13.4%) and trauma patients (13.7%). The nursing workload was higher in Hospital 1, where in most cases each nurse takes care of more than two patients. With regard to the physicians’ workload, it was significantly higher in Hospital 1. It is in this centre that most frequently a physician treats more than three patients (36%). In terms of patient severity on the day of the administration of the AASTRE, the only differences found were in the SOFA subgroup <4 (more prevalent in Hospital 1, 67.1%) and in the SOFA subgroup 4–7 (more prevalent in Hospital 2, 32.7%). Finally, in respect of length of stay in the ICU on the day of the AASTRE, in Hospital 1 there was a significant predominance of patients whose length of stay was less than seven days (54%), the rest of the periods considered were significantly more prevalent in Hospital 2 except the period of ≥21 days, which was virtually identical in both hospitals.

**Table 2:** Distribution of the type and severity of patient disease/condition, staffing ratios and length of stay on the day of evaluation

	Global		Hospital 1		Hospital 2		*p*
	*N*	%	*N*	%	*N*	%	
Patient type							<0.0001
Medical	570	47.0	242	57.6	328	41.3	
Neurosurgery	118	9.7	12	2.9	106	13.4	
Surgical	397	32.7	146	34.8	251	31.6	
Traumatic	129	10.6	20	4.7	109	13.7	
PNR							<0.0001
≤ 2:1	787	64.8	16	3.8	771	97.1	
>2:1	427	35.2	404	96.2	23	2.9	
PPR							<0.0001
≤2:1	143	11.8	29	6.9	114	14.4	
2–3:1	756	62.3	240	57.1	516	65.0	
>3:1	315	25.9	151	36.0	164	20.6	
SOFA							0.001
<4	723	59.6	282	67.1	441	55.6	
4–7	357	29.3	97	23.1	260	32.7	
8–12	110	9.1	31	7.4	79	9.9	
≥12	24	2.0	10	2.4	14	1.8	
Length of stay							<0.0001
<7	532	43.8	227	54.0	305	38.4	
7–14	237	19.5	62	14.8	175	22.0	
14–21	146	12.0	29	6.9	117	14.8	
≥21	299	24.7	102	24.3	197	24.8	

*PNR* patient:nurse ratio, *PPR* patient:physician ratio, *SOFA* Sequential Organ Failure Assessment

### Structure indicators

Perception of safety culture: The response rate to the perception of safety culture questionnaire that had been administered to 71 professionals was 94.4% (in the initial period), 66.6% (in the intermediate period) and 70.4% (in the final period). A progressive increase was observed in positive responses in the Safety Climate item throughout the study period (*p* < 0.0001) in the safety culture perception survey. No significant changes were observed in the other items (Table 3).

**Table 3:** Safety culture survey results in Hospital 2

Dimensions	Start of study *N* = 67 Positive responses, %	Intermediate period *N* = 46 Positive responses, %	End period *N* = 48 Positive responses, %	*p*
Atmosphere in place of work	74.5	67.4	73.4	NS
Relations with colleagues	64.2	59.8	60.9	NS
Organization and management of the service and the hospital	40.8	35.5	42.2	NS
Safety climate	*58.3*	*61.1*	*69.8*	*p* *<* *0.0001*
Work conditions	45.6	37.9	49.5	NS
Recognition of stress level	49.4	43.9	47.3	NS
Total	*56.1*	*53.8*	*60.2*	*p* *=* *0.005*

*NS* no significant differences

Italic text: significant differences

Implementation or updating of protocols and/or procedures: The use of the AASTRE was associated with changes in sedation/analgesia and weaning protocols at both hospitals. It is also noteworthy that in the two hospitals of the study, the use of AASTRE motivated the creation of a new procedure of the prescription and review of monitoring and mechanical ventilation (MV) alarms.

### Process indicators

 The overall IPR-AASTRE-G were 6.7%. Table 4 shows the distribution of patients evaluated for each measure and improvement proportion related to the AASTRE (IPR-AASTRE), and their evolution throughout the study period. Twelve of the 37 measures (32.4%) had IPR-AASTRE >10%. Some are included in the bundles to prevent VAP (evaluation of the level of sedation and pain in the sedated patient, semi-recumbent position) and CRB (daily assessment of catheter needs); others in the good medical practice guidelines (verification of alveolar pressure in patients with acute respiratory failure, assessment of acute renal failure and artificial nutrition) and in the basic safety measures (appropriate treatment prescription, review of MV or monitor alarms, patient identification).

**Table 4:** Distribution of evaluated patients for every measure and improvement proportion related to the AASTRE (IPR-AASTRE)

	Evaluated patients (IPR-AASTRE, %) Total	Evaluated patients (IPR-AASTRE, %) Quarter 1	Evaluated patients (IPR-AASTRE, %) Quarter 2	Evaluated patients (IPR-AASTRE, %) Quarter 3	*p*
Block 1. Mechanical ventilation					
1. Alveolar pressure limit	124 (26.6)	71 (26.8)	20 (45.0)	33 (15.2)	0.12
2. Mechanical ventilation alarms	398 (31.2)	155 (34.2)	119 (33.6)	124 (25.0)	0.001
3. Tolerance to spontaneous ventilation	175 (1.1)	59 (1.7)	56 (0.0)	60 (1.7)	0.62
4. Suitable current volume	336 (1.5)	17 (2.6)	107 (0.9)	112 (0.9)	0.47
Block 2. Haemodynamics					
5. Monitor alarms	557 (21.5)	223 (23.3)	142 (23.9)	192 (17.7)	<0.0001
6. Water balance and fluid adjustment	557 (1.1)	223 (0.5)	142 (0.7)	192 (2.8)	0.36
7. Adequate haemodynamic monitoring	556 (0.4)	223 (0.0)	141 (0.7)	192 (0.5)	0.49
8. Fluid therapy and amines adjustment according to monitoring	93 (1.1)	40 (0.0)	19 (0.0)	34 (2.9)	0.41
Block 3. Renal function and CRRT					
9. Acute renal failure assessment	654 (11.8)	280 (14.6)	160 (10.6)	214 (8.9)	0.03
10. CRRT treatment prescription	27 (3.7)	13 (0.0)	6 (0.0)	8 (12.5)	0.29
11. CRRT monitoring	28 (0.0)	14 (0.0)	6 (0.0)	8 (0.0)	Not calculable
Block 4. Sedation/analgesia					
12. Evaluation of sedation level and pain of sedated patient	199 (11.6)	86 (15.1)	61 (9.8)	52 (7.7)	0.30
13. Pain assessment in non-sedated patient	396 (8.6)	137 (13.1)	110 (5.5)	149 (6.7)	0.19
14. Oversedation prevention	132 (9.8)	65 (10.8)	42 (2.4)	25 (20.0)	0.16
Block 5. Treatment (1)					
15. Check drug allergies and intolerances in patient’s medical history	623 (4.8)	268 (4.1)	161 (6.8)	194 (1.6)	0.10
16. Correct prescription of daily treatment orders	623 (4.8)	268 (5.6)	161 (4.9)	194 (3.6)	0.66
17. Adequate indication and dosage of the prescribed medication	622 (3.9)	268 (4.9)	160 (3.8)	194 (2.6)	0.37
18. Prescribed treatment administered correctly. Verbal orders	623 (1.9)	268 (3.4)	161 (0.0)	194 (1.6)	<0.0001
Block 6. Treatment (2)					
19. Prevention of thromboembolic disease	549 (4.9)	249 (7.2)	146 (3.4)	154 (2.6)	0.04
20. Prophylaxis of gastrointestinal haemorrhage	587 (0.3)	266 (0.4)	159 (0.0)	162 (0.6)	0.63
21. Control of hyperglycaemia	588 (1.4)	264 (1.5)	160 (1.3)	164 (1.2)	0.85
22. Assessment of the antibiotic treatment	436 (2.3)	195 (3.1)	119 (0.8)	122 (2.5)	0.43
23. Appropriate transfusion	554 (0.2)	233 (0.0)	157 (0.6)	164 (0.0)	0.49
Block 7. Techniques and tests					
24. Checking of X-ray slides	539 (1.5)	186 (1.6)	165 (2.4)	188 (0.5)	0.34
25. Daily assessment of the need for catheters	624 (16.8)	225 (16.0)	183 (19.7)	216 (15.3)	0.46
Block 8. Nutrition					
26. Monitoring of enteral nutrition	487 (24.6)	241 (27.0)	117 (27.4)	129 (17.8)	0.11
27. Daily assessment by parenteral nutrition team	78 (23.1)	24 (4.2)	25 (24.0)	29 (37.9)	0.04
Block 9. Nursing care					
28. Verification of endotracheal tube cuff pressure	436 (0.5)	195 (0.0)	127 (0.8)	114 (0.9)	0.58
29. Oral hygiene with chlorhexidine (0.12–0.2%)	476 (0.4)	217 ((0.5)	138 (0.7)	121 (0.0)	0.52
30. Daily assessment of the risk of developing pressure ulcers	563 (12.3)	282 (18.1)	151 (6.6)	130 (6.2)	<0.0001
31. Daily assessment of the protective measures for the safe handling of the patient	557 (1.1)	281 (1.1)	148 (1.4)	128 (0.8)	0.47
32. Semi-recumbent position	433 (21.7)	196 (20.9)	123 (24.4)	114 (20.2)	0.48
Block 10. Structure					
33. Unequivocal patient identification	594 (12.5)	243 (14.4)	190 (12.1)	161 (9.9)	0.42
34. Patient clinical information properly structured in the medical history	592 (24.8)	243 (26.3)	189 (29.1)	160 (17.5)	0.06
35. Life-sustaining treatment limit sheet updated	82 (9.8)	30 (10.0)	22 (4.5)	30 (13.3)	0.44
36. Correct position of bed rails	584 (0.3)	240 (0.8)	185 (0.0)	159 (0.0)	0.24
37. Information to family members	592 (0.2)	242 (0.4)	189 (0.0)	161 (0.0)	0.46

*CRRT* continuous renal replacement techniques

Only six steps (verification of MV or monitor alarms, proper administration of the prescribed treatment, assessment of acute renal failure and the risk of developing pressure ulcers and prevention of thromboembolic disease) showed a progressive decrease in the IPR-AASTRE throughout the study period. In addition, in one measure (“daily assessment by parenteral nutrition team”), a significant increase was seen in IPR-AASTRE as it was assessed during the different four-month periods.

Table 5 shows the impact of the independent variables selected (type of patient, staffing ratio, severity and length of stay) in the IPR-AASTRE-B. The high PNR was associated with a higher IPR-AASTRE in the MV and haemodynamics blocks. The SOFA was associated independently with a higher IPR-AASTRE-B in four blocks. Finally, the length of stay was significantly inversely associated with the IPR-AASTRE-B of the techniques and tests and treatment blocks.

**Table 5:** Variables related to the utility of the AASTRE (multivariate analysis)

	Ratio Patients:nurses OR (95% CI)	Ratio Patients: physicians OR (95% CI)	SOFA OR (95% CI)	Patient type OR (95% CI)	Length of stay OR (95% CI)
Mechanical ventilation	*2.6 (1.1*–*6.7)*	0.9 (0.7–1.0)	*1.4 (1.1*–*1.7)*	1.1 (1.0–1.4)	0.9 (073–1.0)
Haemodynamics	*2.9 (1.2*–*7.4)*	0.9 (0.6–1.3)	0.9 (0.7–1.3)	0.9 (0.8–1.2)	0.9 (0.8–1.2)
Renal function/CRRT	0.3 (0.0–1.4)	0.9 (0.6–1.4)	*2.0 (1.5*–*2.5)*	1.1 (0.9–1.4)	0.9 (0.8–1.1)
Sedation and analgesia	0.9 (0.2–3.5)	0.9 (0.5–1.3)	1.2 (0.8–1.7)	1.0 (0.8–1.3)	0.9 (0.7–1.1)
Treatment 1	0.5 (0.1–2.7)	1.4 (0.9–2.0)	*1.4 (1.1*–*1.8)*	0.9 (0.8–1.1)	*0.8 (0.6*–*0.9)*
Treatment 2	1.5 (0.2–11.1)	1.5 (0.9–2.5)	1.1 (0.8–1.7)	0.9 (0.7–1.3)	*0.6 (0.4*–*0.8)*
Techniques and tests	1.3 (0.4–3.6)	0.6 (0.4–1.9)	0.9 (0.7–1.2)	0.9 (0.8–1.2)	*0.7 (0.5*–*0.9)*
Nutrition	0.5 (0.1–2.5)	1.2 (0.8–1.6)	0.9 (0.8–1.3)	1.1 (0.9–1.3)	0.9 (0.8–1.1)
Nursing care	1.2 (0.5–3.2)	1.1 (0.8–1.4)	*1.4 (1.1*–*1.7)*	0.9 (0.8–1.1)	0.9 (0.8–1.2)
Structure	1.0 (0.4–2.5)	1.0 (0.7–1.2)	1.2 (1.0–1.5)	1.1 (0.9–1.3)	0.9 (0.8–1.1)

*CRRT* continuous renal replacement techniques

Italic text: significant OR

### Outcome indicators

The use of the AASTRE was associated with a significant decline in the VAP rate. No significant impact on average stay, mortality and CRB rate was observed (Table 6).

**Table 6:** Outcome indicators

Year	Hospital 1			Hospital 2		
	2012	2013	*p*	2012	2013	*p*
Number of patients	967	1018		939	927	
APACHE II	10.4	11.2	0.39	14.9	14.4	0.72
ICU average LOS (days)	3.3	4.0	0.09	7.7	7.9	0.07
ICU gross mortality (%)	4.0	5.1		19.0	16.1	
ICU standardized mortality by APACHE 2012 (%)	4.0	4.3	0.23	19.0	16.5	0.15
VAP rate (No. of VAP episodes/1000 days MV)	4.2	0.9		7.9	4.0	
IDR VAP_2013/2012_ (95% confidence interval)	0.2 (0.05–0.9)		0.02	0.5 (0.3–0.9)		0.03
CRB rate (No. of CRB episodes/1000 days of central venous catheter)	3.9	1.5		2.8	4.8	
CRB IDR_2013/2012_ (95% confidence interval)	0.4 (0.1–1.2)		0.42	1.7 (0.7–3.9)		0.10

*N* number, *VAP* ventilator-associated pneumonia, *MV* mechanical ventilation, *CRB* central venous catheter-related bacteraemia , *IDR* incidence density ratio

## Discussion

Checklists have been proposed as tools to ensure that essential components of care are not omitted [15]. However, this is the first multicentre study to analyse the impact of real-time random safety audits on quality indicators in the critical patient. An improvement is seen in indicators of structure (safety climate, clinical protocols and healthcare procedures), process (better adherence to good clinical practice guidelines) and outcome (decline in the rate of VAP) [16]. These data support a way to improve health care for the critical patient by means of the AASTRE tool whose use is feasible as shown in the pilot study published previously by our group [10].

### Structure indicators

The use of the AASTRE was associated with an improvement in the safety climate. An association has been described between a better safety climate and outcome [17], average stay [18] or adverse events [19]. Although other authors have not demonstrated that checklists improve communication and teamwork [20], the impact of the AASTRE on the safety climate could be the result of improved communication in clinical practice, as described in other tools [21].

The guidelines require local adaptation via local protocols to enable their effective, safe and efficient use [22, 23]. The AASTRE were associated with the need to renew sedation/analgesia and weaning protocols. This occurs as a natural consequence when verifying the safety measures using AASTRE reflexively and at the bedside. This highlights the need to update local protocols in accordance with the latest sources of scientific knowledge. Difficulties for adherence have been described [24, 25] in both protocols. The AASTRE allow evaluating adherence to protocols and can promote their regular updating. The AASTRE have improved safety in relation to monitoring and mechanical ventilation alarms through the creation of specific protocols.

While the beneficial effect of the introduction of protocols in clinical practice has been discussed [26, 27], most studies acknowledge that they are useful although more patently in hands of inexperienced healthcare providers or suboptimal work environments. In such malfunctioning environments, they help but may hinder the performance and progress of the health professional, reducing their autonomy [28]. In fact, in a study published recently, no effect of the protocols was observed in the outcome [29]. Therefore, AASTRE promote much needed professional autonomy but invites reflection as to decisions at the bedside. And this reflection leads to the updating of protocols even though a direct improvement in the results is not guaranteed.

### Process indicators: IPR-AASTRE

Health care requires many more scientifically sound process measures than are currently available. The AASTRE are process indicators since they evaluate the degree of adherence to scientific evidence [30]. They allow measuring the gap between the indication of therapies that have proven effective within human clinical research and the real safe and effective use of these therapies in routine clinical practice.

The failure to ensure that patients receive recommended medical care is supported by high-quality clinical research evidence. This type of safety and quality problem can be effectively addressed with knowledge translation tools [23].

The fact that 12 of the 37 measures considered (32.4%) had IPR-AASTRE >10% shows the ability of the AASTRE to modify essential aspects of clinical practice and improve adherence to evidence-based guidelines, a priority in health care [11, 23, 31]. Hopefully, through organizational learning, this effect could be maintained over time [32]. In this regard, some authors [33] have described the ability of checklists to maintain adherence to good clinical practice guidelines achieving close to 100% compliance for semi-recumbent position or suitable sedation. However, in our study, these measures scored IPR-AASTRE of 21.7 and 11.6%, indicating that if the intervention (AASTRE) had not been implemented, the measure would not have been carried out in a large percentage of patients. Also, the evaluation of another essential measure as is the assessment of catheter needs, in our experience, was corrected in 16.8% of evaluations. The fact that IPR-AASTRE utility is maintained over time may be related to the complexity of ICU clinical activity. In this regard, the AASTRE act as a tool that redirects healthcare activity towards essential aspects of care, regardless of the environmental situation. However, in our study six measures showed a significant decrease along the four-month periods analysed, indicating that this tool can also help systematize healthcare and organizational learning. Nevertheless, in the case of measures with a gradually ascending IPR-AASTRE, it should be verified (as occurs in this study with the measure “daily assessment by parenteral nutrition team”) that the lack of adherence to the recommendations can be accounted for by causes from outside the work team implementing AASTRE (a problem of communication with other agents involved in treating the patient, as might occur in this case with professionals of the Pharmacy Service who are responsible for monitoring hospital parenteral nutrition, for example).

The AASTRE have proven to be more effective in more serious patients, in the early days of admission and in increased workload environments. These findings are consistent with the data published by our group previously [11]. Without interrupting the work flow, aspects of severe patient care are recalled and their definitive inclusion into treatment is left to the discretion of the senior physician responsible of the patient, according to the indication:risk ratio.

### Outcome indicators

The concept of care bundling and its efficacy in improving clinical outcomes are also supported in the literature [34]. Our results show a significant decrease in VAP influencing three of the recommendations established to prevent this type of adverse event (assessment of sedation level, semi-recumbent position and prophylaxis of deep venous thrombosis). Dubose et al. [35] described this effect at a trauma ICU through a checklist of VAP bundle measures. However, in our study, the AASTRE had no impact on mortality and CRB. Probably, to demonstrate an impact on the rate of CRB requires influencing other aspects such as catheter insertion and maintenance [36].

In the critical patient, no study has managed to associate the use of safety checklists with a decrease in mortality [15]. Recently, in a study of Brazilian ICUs [37], the introduction of daily checklists, goal setting and clinician prompting did not decrease in-hospital mortality or other clinical outcomes. Despite being a study with a considerable sample size, some organizational and methodological aspects could render the results unreproducible outside that environment. For example, the health system is not comparable to the European one as regards cultural aspects and the organization of work teams. Moreover, standardized mortality is high and the number of patients recruited in each ICU was relatively low. In addition, important methodological aspects such as the period of analysis (just 4 months in that study), the definition of the measures, the eligibility of the patients and training in the use of the tools are different in the two studies. Nevertheless, the most distinguishing factor between the studies is the role of the prompter. According to the authors of the Brazilian study, the feedback of the clinician with the prompter was carried out later in the day. In our study, this is one of the keys of our methodology, the prompter interacts at the bedside during healthcare activity, immediately after the daily clinical rounds, acting as a catalyser of the transfer of knowledge, thus improving adherence to scientific proof. In any case, we are aware that a single intervention, albeit cross-cutting, never has a definitive impact on patient prognosis. Moreover, using mortality as an outcome measure requires larger samples and risk adjustment for fair comparison among providers and organizations [38].

There are limitations to this study. (1) Only two ICUs have participated. Moreover, their participation in the design of the AASTRE tool, the experience gained by the research team from the pilot study and the development of the culture of the continuous improvement of the quality of care that is underlying in the participating centres may mean that it is not possible to extrapolate the results to other ICUs. (2) The Hawthorne effect, a performance gain resulting from the knowledge of being observed, is difficult to distinguish from those resulting from the intervention. (3) The perception of safety culture was investigated only at one centre. (4) Sample size was not initially calculated to investigate the impact of the AASTRE on mortality or nosocomial infection rates. (5) The study design does not include a control group, since the random selection of the patients evaluated in the safety audits does not allow this. (6) Having demographic data of the patient populations attended to during the study period, of the quantitative evaluation of the Nursing workload and of the incidence of adverse events may have helped establish more precise analysis of the data and of the impact of AASTRE (Additional file 1).

## Conclusions

In conclusion, our results suggest that the AASTRE were associated with improved structure, process and outcome indicators. In addition, this tool allows simultaneously translating medical evidence to clinical practice, reducing errors of omission, and also allows assessing quality through process indicators.

## Additional file

**Additional file 1.** Database of AASTRE study.

## Abbreviations

ICU: intensive care unit
AASTRE: Análisis Aleatorios de Seguridad en Tiempo Real
CRRT: continuous renal replacement techniques
SCS: Safety Climate Survey
SAQ-ICU: Safety Attitude Questionnaire-ICU model
PNR: patient:nurse ratio
PPR: patient:physician ratio
SOFA: Sequential Organ Failure Assessment
NS: no significant differences
IPR-AASTRE: improvement proportion related to the AASTRE
IPR-AASTRE-G: improvement proportion related to the AASTRE globally
IPR-AASTRE-B: improvement proportion related to the AASTRE for each block of variables
MV: mechanical ventilation
CRB: central venous catheter-related bacteraemia
VAP: ventilator-associated pneumonia (VAP)
CVC: central venous catheter

## References

[1] Curtis JR, Cook DJ, Wall RJ, Angus DC, Bion J, Kacmarek R (2006). Intensive care unit quality improvement: a how-to guide for the interdisciplinary team. Crit Care Med.

[2] Cabana MD, Rand CS, Powe NR, Wu AW, Wilson MH, Abboud PA (1999). Why don’t physicians follow clinical practice guidelines? A framework for improvement. JAMA..

[3] Ilan R, Fowler RA, Geerts R, Pinto R, Sibbald WJ, Martin CM (2007). Knowledge translation in critical care: factors associated with prescription of commonly recommended best practices for critically ill patients. Crit Care Med..

[4] Ursprung R, Gray JE, Edwards WH, Horbar JD, Nickerson J, Plsek P (2005). Real time patient safety audits: improving safety every day. Qual Saf Health Care.

[5] Byrnes MC, Schuerer DJE, Schallom ME, Sona CS, Mazuski JE, Taylor BE (2009). Implementation of a mandatory checklist of protocols and objectives improves compliance with a wide range of evidence-based intensive care unit practices. Crit Care Med.

[6] Bergs J, Lambrechts F, Simons P, Vlayen A, Marneffe W, Hellings J (2015). Barriers and facilitators related to the implementation of surgical safety checklist: a systematic review of the qualitative evidence. BMJ Qual Saf.

[7] Lee L, Girish S, Van den Berg E, Leaf A (2009). Random safety audits in the neonatal unit. Arch Dis Child Fetal Neonatal Ed.

[8] Leape LL, Berwick DM, Bates DW (2002). What practices will most improve safety?. Evidence-based medicine meets patient safety. JAMA.

[9] Weiss CH, Moazed F, McEvoy CA, Singer BD, Szleifer I, Amaral LA (2011). Prompting physician to address a daily checklist and process of care and clinical outcomes. Am J Respir Crit Care Med.

[10] Sirgo Rodríguez G, Olona Cabases M, Martin Delgado MC, Esteban Reboll F, Pobo Peris A, Bodí Saera M, ART-SACC Study Experts (2014). Audits in real time for safety in critical care: definition and pilot study. Med Intensiva.

[11] Bodí M, Olona M, Martín MC, Alceaga R, Rodríguez JC, Corral E (2015). Feasibility and utility of the use of real time random safety audits in adult ICU patients: a multicentre study. Intensive Care Med.

[12] Gutiérrez-Cía I, de Cos PM, Juan AY, Obón-Azuara B, Alonso-Ovies Á, Martin-Delgado MC (2010). Perception of safety culture in Spanish intensive care units. Med Clin.

[13] Mermel LA, Allon M, Bouza E, Graven DE, Flynn P, O’Grady NP (2009). Clinical practice guidelines for the diagnosis and management of intravascular catheter-related infections: 2009 update by the Infectious Diseases Society of America. Clin Infect Dis.

[14] Klompas M, Kleinman K, Khan Y, Evans RS, Lloyd JF, Stevenson K (2012). Rapid and reproducible surveillance for ventilator-associated pneumonia. Clin Infect Dis.

[15] Thomassen Ø, Storesund A, Søfteland E, Brattebø G (2014). The effects of safety checklist in medicine: a systematic review. Acta Anaesthesiol Scand.

[16] Mittman BS (2004). Creating the evidence base for quality improvement collaborative. Ann Intern Med.

[17] Singer S, Gaba D, Geppert J, Sinaiko AD, Howard SK, Park KC (2003). The culture of safety: results of an organization-wide survey in 15 California hospitals. Qual Saf Health Care.

[18] Huang DT, Clermont G, Kong L, Weissfeld LA, Sexton JB, Rowan KM (2010). Intensive care unit safety culture and outcomes: a US multicenter study. Int J Qual Health Care.

[19] Valentin A, Schiffinger M, Steyrer J, Huber C, Strunk G (2013). Safety climate reduces medication and dislodgement errors in routine intensive care practice. Intensive Care Med.

[20] Böhmer AB, Kindermann P, Schwanke U, Bellendir M, Tinschmann T, Schmidt C (2013). Long-term effects of a perioperative safety checklist from the viewpoint of personnel. Acta Anaesthesiol Scand.

[21] Randmaa M, Mårtensson G, Leo Swenne CL, Engström M (2014). SBAR improves communication and safety climate and decreases incident reports due to communication errors in an anaesthetic clinic: a prospective intervention study. BMJ Open.

[22] Roffey P, Thangathurai D (2011). Increased use of protocols in ICU settings. Intensive Care Med.

[23] Needham DM (2010). Patient safety, quality of care, and knowledge translation in the intensive care unit. Respir Care.

[24] Sneyers B, Laterre PF, Perreault MM, Wouters D, Spinewine A (2014). Current practices and barriers impairing physicians’ and nurses’adherence to analgo-sedation recommendations in the intensive care unit–a national survey. Crit Care.

[25] Rose L, Dainty KN, Jordan J (2014). Weaning from mechanical ventilation: a scoping review of qualitative studies. Am J Crit Care.

[26] Soares M, Bozza FA, Angus DC, Japiassú AM, Viana WN, Costa R (2015). Organizational characteristics, outcomes, and resource use in 78 Brazilian intensive care units: the ORCHESTRA study. Intensive Care Med.

[27] Isherwood P (2016). Response to: Protocols: help for improvement but beware of regression to the mean and mediocrity. Intensive Care Med.

[28] Girbes AR, Robert R, Marik PE (2015). Protocols: help for improvement but beware of regression to the mean and mediocrity. Intensive Care Med.

[29] Sevransky JE, Checkley W, Herrera P, Pickering BW, Barr J, Brown SM, United States Critical Illness and Injury Trials Group-Critical Illness Outcomes Study Investigators (2015). Protocols and hospital mortality in critically ill patients: the USA Critical Illness and Injury Trials Group Critical Illness Outcomes Study. Crit Care Med.

[30] Pronovost P, Holzmueller CG, Needham DM, Sexton JB, Miller M, Berenholtz S (2006). How will we know patients are safer? An organization-wide approach to measuring and improving safety. Crit Care Med.

[31] Kiyoshi-Teo H, Cabana MD, Froelicher ES, Blegen MA (2014). Adherence to institution-specific ventilator-associated pneumonia prevention guidelines. Am J Crit Care.

[32] Wadhwani V, Shillingford A, Penford G, Thomson MA (2011). Random safety audits for improving standards in the neonatal unit. Arch Dis Child Fetal Neonatal Ed.

[33] Teixeira PGR, Inaba K, DuBose J, Melo N, Bass M, Belzberg H (2013). Measurable outcomes of quality improvement using a daily quality rounds checklist: two-year prospective analysis of sustainability in a surgical intensive care unit. J Trauma Acute Care Surg.

[34] Resar R, Pronovost P, Haraden C, Simmonds T, Rainey T, Nolan T (2005). Using a bundle approach to improve ventilator care processes and reduce ventilator-associated pneumonia. Jt Comm J Qual Patient Saf.

[35] Dubose J, Teixeira PG, Inaba K, Lam L, Talving P, Putty B (2010). Measurable outcomes of quality improvement using a daily quality rounds checklist: one-year analysis in a trauma intensive care unit with sustained ventilator-associated pneumonia reduction. J Trauma.

[36] Hsu YJ, Marsteller JA (2016). Influence of the comprehensive Unit-based Safety Program in ICUs: evidence from the keystone ICU Project. Am J Med Qual.

[37] Cavalcanti AB, Bozza FA, Machado FR, Salluh JI, Campagnucci VP, Vendramim P, Writing Group for the CHECKLIST-ICU Investigators and the Brazilian Research in Intensive Care Network (BRICNet) (2016). Effect of a quality improvement intervention with daily round checklists, goal setting, and clinician prompting on mortality of critically ill patients: a randomized clinical trial. JAMA.

[38] Weled BJ, Adzhigirey LA, Hodgman TM, Brilli RJ, Spevetz A, Kline AM (2015). Critical care delivery: the importance of process of care and ICU structure to improved outcomes: an update from the American College of critical care medicine task force on models of critical care. Crit Care Med.

